# Rate of Post-Fertilization Mitotic Activity Predicts Embryonic Competence via Next Generation Sequencing: An Analysis of 39,301 Cleavage Stage Embryos

**DOI:** 10.5935/1518-0557.20210051

**Published:** 2021

**Authors:** Jenna Friedenthal, Stephanie Pan, Dmitry Gounko, Christine Briton-Jones, Joseph Lee, Alan Copperman

**Affiliations:** 1 Department of Obstetrics, Gynecology, and Reproductive Science. Icahn School of Medicine at Mount Sinai. New York, NY 10029, USA; 2 Reproductive Medicine Associates of New York. New York, NY 10022, USA

**Keywords:** next generation sequencing, cleavage stage embryos, rapid cell division, preimplantation genetic testing, euploidy

## Abstract

**Objective:**

To investigate the association between cleavage stage development, embryonic competence, and euploidy in patients undergoing in vitro fertilization (IVF) with subsequent next generation sequencing.

**Methods:**

The retrospective cohort study included patients at an academic fertility center who underwent IVF with at least one cleavage stage embryo from 2016 to 2019. Embryos were analyzed as slow (<6 cells), intermediate (6-8 cells), or fast (>8 cells); day 3 cell count was also analyzed as a continuous variable. Primary outcomes were blastulation rate, biopsied blastocyst rate, and euploid rate. Odds of blastulation, biopsy, and euploidy were also calculated. Additionally, we modeled the predicted probability of an embryo reaching blastulation, biopsy, and euploidy based on cleavage stage development.

**Results:**

When compared with intermediate and slow cohorts, fast cleaving embryos had significantly higher rates of blastulation (82.70% *vs*. 75.13 *vs*. 42.48%), biopsy (55.04% vs. 44.00% vs. 14.98%), and euploidy (50.65% *vs*. 47.93% *vs*. 48.05%). After adjustment for covariates, there was a significant association between cleavage stage development and odds of blastulation (OR 1.38, 95% CI 1.29-1.48), biopsy (OR 1.42, 95% CI 1.34-1.51), and euploidy (OR 1.08, 95% CI 1.01-1.17). Finally, we observed significant associations between cleavage stage development and predicted probability of reaching blastulation (OR 1.29, 95% CI 1.27-1.32), biopsy (OR 1.24, 95% CI 1.22-1.26), and euploidy (OR 1.02, 95% CI 1.01-1.04).

**Conclusions:**

Cleavage stage embryos with greater mitotic activity perform as well as or better than intermediate or slower cleaving embryos. Rapidly cleaving embryos have high rates of euploidy and significant clinical potential.

## INTRODUCTION

The ability to define markers of embryonic competence in order to enhance cycle outcome success is one of the most important aspects of assisted reproductive technology (ART) treatment. Previous research has suggested that early development to the cleavage stage may be a strong predictor of outcomes ^(Bos-Mikich *et al*., 2001)^. For years, rapid cell division of the early stage embryo was thought to be "chaotic," and cleavage stage embryos with more than eight cells were thought to have poor developmental potential ^(Alikani *et al*., 2000)^. However, data is conflicting, as recent published work has demonstrated that rapidly dividing cleavage stage embryos have a high likelihood of blastulation ^(Luna *et al*., 2008; Lee *et al*., 2012)^.

Additionally, it is well established that the majority of first trimester losses are due to embryonic aneuploidy ^(Petracchi *et al*., 2009)^. With the development of extended culture to blastocyst and the ability to perform trophectoderm (TE) biopsy, pre-implantation genetic testing for aneuploidy (PGT-A) has become the modern standard to distinguish chromosomally normal and abnormal embryos, with the goal of improving pregnancy rates and reducing miscarriage rates. Prior work has attempted to correlate cleavage stage development with embryonic aneuploidy. However, these studies used older technologies (e.g. fluorescence in situ hybridization (FISH), SNP array, and/or array comparative genomic hybridization, or aCGH) ^(Luna *et al*., 2008; Campbell *et al*., 2013; Anderson *et al*., 2004; Magli *et al*., 1998)^. To date, there has yet to be a study correlating cleavage stage cell division with ploidy status as diagnosed by next generation sequencing (NGS). NGS has emerged as the best-in-class platform for PGT-A and has become increasingly popular due to its ability to perform high throughput sequencing with decreased cost ^(Munné & Wells, 2017)^. NGS is also able to better identify segmental aneuploidies, polyploidy, unbalanced translocations, and mosaicism as compared with other PGT-A platforms ^(Maxwell *et al*., 2016; Friedenthal *et al*., 2018)^. Thus, the goal of our study was to assess the relationship between rate of cleavage stage development, embryonic competence, and euploid rate as diagnosed by NGS.

## MATERIALS AND METHODS

### Study design and patient population

This study was approved by an academic Institutional Review Board (IRB# 18-00452). The retrospective cohort study included patients from a single academic center who underwent controlled ovarian stimulation, in vitro fertilization (IVF) with intracytoplasmic sperm injection (ICSI), and had at least one embryo that reached cleavage stage from 2016 to 2019. Exclusion criteria included cases involving multiple biopsies on the same blastocyst, patients who utilized donor oocytes, or patients with incomplete demographic data. Additionally, as per our center's standard operating procedure, embryos with ≤ 3 cells on day 3 are discarded and were excluded from this study's analysis.

Demographic baseline clinical data were collected, including patient age, body mass index (BMI), and AMH level (ng/mL). Percent embryo fragmentation and cell count were evaluated on day 3 of development. Additionally, the time of embryo assessment was computed as the duration (in hours) between the completion of ICSI on day 0 and the time of assessment on day 3 by our embryologists. The mean duration in culture was determined over the study population and used to calculate an adjusted cleavage stage cell count as described in our statistical analysis.

### Stimulation protocol and laboratory procedures

Controlled ovarian stimulation was performed as described previously ^(Sekhon *et al*., 2018)^. When at least two follicles reached ≥18mm in diameter, trigger of final maturation was performed using either recombinant HCG alone, or 40 IU of a GnRH agonist in combination with 1000IU HCG. Subsequently, patients underwent oocyte retrieval under transvaginal ultrasonographic guidance. All oocytes that reached the metaphase II stage (MII) underwent ICSI approximately 4-6 hours after retrieval. Those embryos in which fertilization had been confirmed on day 1 of development were then evaluated on day 3 for cleavage stage development. Embryos were then cultured to the blastocyst stage and subsequently underwent assisted hatching. Blastocyst TE biopsies were then performed on day 5, 6, or 7 of development if an embryo reached a morphologic grade of ≥4CC (Modified Gardner score). Detailed description of our embryo culture and TE biopsy techniques have been described elsewhere ^(Hernandez-Nieto *et al*., 2019)^. PGT-A was performed was performed using NGS at partnering genetics laboratories.

### Outcomes

Our primary outcome was euploid rate (ER), calculated for each cycle as the number of embryos diagnosed as euploid by trophectoderm (TE) biopsy for NGS divided by the total number of blastocysts that were biopsied. All embryos were screened using NGS for PGT-A. Secondary outcomes included blastulation rate and biopsied blastocyst rate. Blastulation rate was calculated as the number of embryos that developed to the blastocyst stage divided by the number of cleavage stage embryos. Biopsied blastocyst rate was calculated as the number of blastocysts that were biopsied for PGT-A divided by the number of cleavage stage embryos. Additionally, we modeled the predicted probability of an embryo reaching blastulation, the predicted probability of an embryo being biopsied, and the predicted probability of an embryo being euploid based on day 3 cell count as described in our statistical analysis.

### Statistical analysis

Cleavage stage cell division was analyzed as both a categorical and a continuous variable. As a categorical variable, embryos that reached the cleavage stage were divided into three cohorts: slow, intermediate, and fast. Adjusted cleavage stage cell counts were calculated by determining the mean time in culture (m) over the sample population and scaling each cell count by the ratio mean/ (time in culture). Slow growing cleavage stage embryos were defined as having < 6 cells. Intermediate developing cleavage stage embryos were defined as having 6-8 cells. Fast developing cleavage stage embryos were defined as embryos with > 8 cells. Demographics and clinical outcomes were described and compared between cleavage stage cohorts using linear and logistic generalized estimating equation (GEE) regression models. In adjusted analyses, the association between cleavage stage cell division and our outcomes were analyzed using logistic multivariable generalized estimating equation (GEE) regression models to account for within-correlation at the patient and cycle level, using the intermediate group as the reference group. Clinically relevant covariates in the model included patient age, body mass index (BMI), anti-Müllerian hormone (AMH) levels, and percent embryo fragmentation. Patient age was grouped as follows according to the Society for Assisted Reproductive Technology (SART): < 35, 35 - 37, 38 - 40, 41 - 42, and > 42 years old. Embryo fragmentation was categorized as follows: <10%, 10-20%, and >20%.

We repeated the analysis of rate of cleavage stage cell division with our primary and secondary outcomes, but treated cell count as a continuous variable. Univariate and multivariable logistic GEE models were performed. Model estimates were utilized to graphically illustrate the predicted probability of progressing to blastocyst, reaching trophectoderm biopsy, and euploidy. Predicted probabilities were stratified by age groups with BMI and AMH fixed at the patient sample mean and percent embryo fragmentation at the < 10% category. Results were reported as odds ratios (ORs) with 95% confidence intervals (95% CIs). A value of *p*<0.05 was considered statistically significant. Statistical analyses were performed with SAS version 9.4 (SAS Institute Inc, Cary, NC).

## RESULTS

A total of 39,301 cleavage stage embryos from 3,522 patients were assessed in the study and met inclusion criteria. Demographics and clinical characteristics are presented and stratified by cleavage stage development (e.g. slow, intermediate, fast) in Table 1. The average patient age was higher in the slow developing cohort compared to intermediate and fast cohorts (36.67±4.48 *vs*. 36.67±4.58 *vs*. 36.23±4.67, *p*<0.0001). AMH, in contrast, was lower in patients with slow developing embryos compared to the other groups. Further, slower developing embryos had, on average, a higher proportion of fragmentation relative to the other groups (8.24%±10.65 *vs*. 4.15%±6.93 *vs*. 2.70%±5.08, *p*<0.0001).

**Table 1.** Demographic and clinical data of embryos from patients undergoing IVF-ICSI from 2016 to 2019.

	Slow (n=6,153 embryos)	Intermediate (n=13,894 embryos)	Fast (n=19,254 embryos)	*p*-value
	Mean ± SD or No. (%)	Mean ± SD or No. (%)	Mean ± SD or No. (%)	
Demographics				
Age (years)	36.67±4.48	36.67±4.58	36.23±4.67	<0.0001
BMI (kg/m^2^)	24.10±4.55	24.07±4.43	24.10±4.44	0.85
AMH (ng/mL)	3.81±4.21	3.82±4.10	4.20±4.80	0.002
Embryo Fragmentation (%)	8.24±10.65	4.15±6.93	2.70±5.08	<0.0001
Clinical Outcomes				
Blastulation Rate	2,614/6,153 (42.48)	10,439/13,894 (75.13)	15,924/19,254 (82.70)	<0.0001
Biopsied Blastocyst Rate	922/6,153 (14.98)	6,113/13,894 (44.00)	10,597/19,254 (55.04)	<0.0001
Euploid Rate	443/922 (48.05)	2,930/6,113 (47.93)	5,367/10,597 (50.65)	0.005

In the adjusted multivariable analysis (Table 2), we observed that fast growing embryos had significantly higher odds of reaching blastulation compared to intermediate or slow developing embryos (*p*<0.0001). Additionally, fast developing embryos had significantly higher odds of reaching trophectoderm biopsy after adjusting for confounders (*p*<0.0001). Finally, fast growing embryos were more likely to be diagnosed as euploid by NGS compared to intermediate or slow growing embryos (*p*=0.03).

**Table 2.** Multivariable GEE Logistic Regression Models with Cleavage Stage Cohorts.

	Slow (N=6,153 embryos)	Intermediate (N=13,894 embryos)	Fast (N=19,254 embryos)	*p*-value
	Adjusted OR (95% CI)*	Reference	Adjusted OR (95% CI)*	
Blastulation Rate	0.28 (0.26, 0.30)	1.0	1.38 (1.29,1.48)	<0.0001
Biopsied Blastocyst Rate	0.25 (0.23, 0.28)	1.0	1.42 (1.34,1.51)	<0.0001
Euploid Rate	0.93 (0.79, 1.08)	1.0	1.08 (1.01,1.17)	0.03

* Adjusted analyses were performed using a multivariable logistic generalized estimation equation (GEE) modeling adjusted embryo cell division speed at day 3 (cleavage stages: slow <6, intermediate 6-8, fast >8) accounting for within-correlation at the patient and cycle level, controlling for patient age, BMI, AMH, and percent embryo fragmentation.

**Figure 1:**
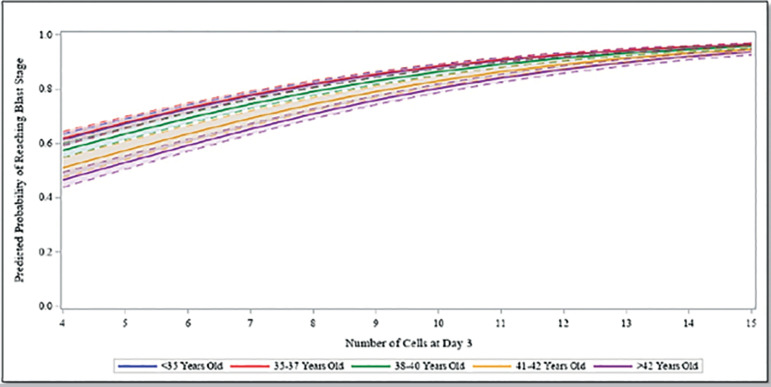
Predicted Probability of Reaching Blastocyst Stage. Predicted probability of reaching blastulation for the number of cells at day 3 by oocyte age group, fixing BMI and AMH using the patient sample mean and fragmentation at <10%.

**Figure 2:**
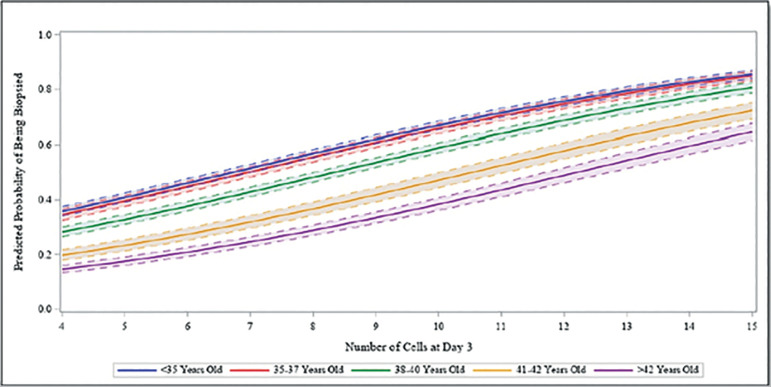
Predicted Probability of Reaching Biopsy. Predicted probability of being biopsied for the number of cells at day 3 by oocyte age group, fixing BMI and AMH using the patient sample mean and fragmentation at <10%.

**Figure 3:**
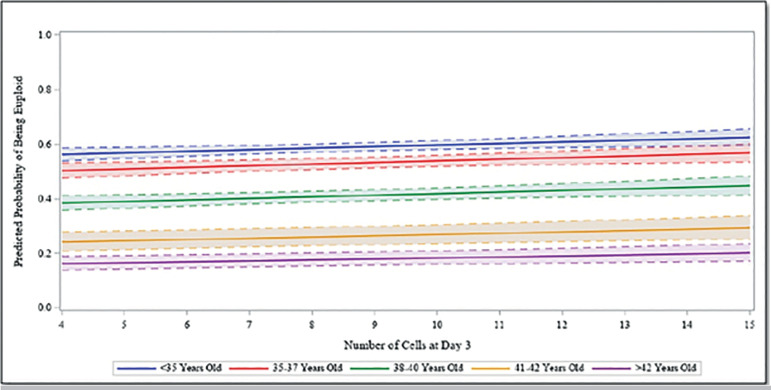
Predicted Probability of Being Euploid. Predicted probability of being euploid for the number of cells at day 3 by oocyte age group, fixing BMI and AMH using the patient sample mean and fragmentation at <10%.

On a continuous scale for cleavage stage cell division, the estimates from the adjusted multivariable analysis are reported in Table 3. After accounting for patient age, BMI, AMH, and percent embryo fragmentation, we observed a significant association between cleavage stage development and the odds of an embryo reaching blastocyst, being biopsied, and being diagnosed euploidy. The odds of an embryo developing to the blastocyst stage was 1.29 times or 29% higher (95% CI 1.27-1.32) for each increase in cell count, controlling for all other covariates. The odds of an embryo reaching biopsy was 1.24 times or 24% higher (95% CI 1.22-1.26) for each increase in cell count, controlling for confounders. Finally, there was a significant association between increasing day 3 cell count and odds of euploidy (*p*=0.007). The predicted probabilities of a cleavage stage embryo developing to the blastocyst stage, reaching trophectoderm biopsy, and being euploid after accounting for other covariates and for each cell count assessed are illustrated in Figures 1-3, respectively. The predicted probability of an embryo reaching each of the outcomes was significantly higher in fast developing embryos compared with intermediate or slow growing cleavage stage embryos, as well as in younger compared with older aged patient age groups.

**Table 3.** Multivariable GEE Logistic Regression Models with Cleavage Stage Embryo Cell Count.

Variables	Blastulation		Blastocyst Biopsied		Euploid	
	Adj. OR (95% CI)*	*p*-value	Adj. OR (95% CI)*	*p*-value	Adj. OR (95% CI)*	*p*-value
Adjusted Day 3 Cell Count	1.29 (1.27, 1.32)	<0.0001	1.24 (1.22, 1.26)	<0.0001	1.02 (1.01, 1.04)	0.007
Fragmentation Percent Low (<10%) Moderate (10-20%) High (>20%)	Reference 0.39 (0.37, 0.42) 0.15 (0.13, 0.18)	<0.0001	Reference 0.38 (0.35, 0.41) 0.10 (0.07, 0.13)	<0.0001	Reference 1.05 (0.93, 1.18) 2.09 (1.26, 3.44)	0.01
Patient Age Group A (< 35) B (35-37) C (38-40) D (41-42) E (>42)	Reference 1.01 (0.91, 1.13) 0.84 (0.76, 0.94) 0.65 (0.57, 0.75) 0.54 (0.49, 0.60)	<0.0001	Reference 0.95 (0.87, 1.04) 0.71 (0.64, 0.77) 0.44 (0.39, 0.50) 0.31 (0.28, 0.34)	<0.0001	Reference 0.79 (0.72, 0.87) 0.49 (0.44, 0.54) 0.25 (0.21, 0.30) 0.15 (0.13, 0.18)	<0.0001
BMI	1.00 (0.99, 1.01)	0.55	0.99 (0.98, 1.00)	0.03	1.01 (1.00, 1.02)	0.12
AMH	1.06 (1.01, 1.11)	0.03	0.96 (0.91, 1.00)	0.09	1.05 (1.00, 1.10)	0.02

* Adjusted analyses were performed using a multivariable logistic generalized estimation equation (GEE) modeling embryo cell division speed at day 3 accounting for within-correlation at the patient and cycle level, controlling for patient age, BMI, AMH, and percent embryo fragmentation.

## DISCUSSION

Our results demonstrated that early mitotic activity is an important marker of genomic competence. Rapid cleavage stage development not only predicted blastulation, but also euploidy as diagnosed by NGS. Patient age played a significant role in predicting outcomes, with patients from younger SART age groups having significantly greater odds of their embryos reaching the blastocyst stage, trophectoderm biopsy, and being euploid than patients from older SART age groups. Additionally, our study demonstrated that embryos that reach the blastocyst stage and are eligible for biopsy have an equal likelihood of being euploid. To our knowledge, this study is the first to utilize NGS for PGT-A to correlate chromosomal copy number with cleavage stage cell division.

Previous work has established the significance of cell count on day 3 as an important predictor of clinical outcomes ^(Racowsky *et al*., 2000)^. Our findings are in accordance with prior studies that have determined that slow growing cleavage stage embryos have reduced embryonic competence. However, our study counters many of the previous findings and governing body suggestions about cleavage stage cell count and reproductive potential ^(Magli *et al*., 2007; Finn *et al*., 2010)^.

It is important to note several differences between these studies and our work. First, unlike prior studies that performed embryo biopsies on day 3 of development, ours utilized trophectoderm biopsy at day 5, 6, or 7. As prior work has demonstrated the detrimental effect of day 3 biopsy when compared with blastocyst biopsy ^(Scott Jr *et al*., 2013)^, utilization of day 3 blastomere biopsy introduces inherent bias to their findings and may significantly alter the clinical potential of the cleavage stage embryos in their studies. Prior studies also had smaller sample sizes relative to the large sample size of our study. Additionally, as much of this work was performed several years prior to the current study, it must be recognized that significant changes have occurred in culture conditions, including changes in media ^(Sunde *et al*., 2016)^, improved laboratory circulation and incubation environments, and changes in oxygen tension which may have impacted their findings ^(Kirkegaard *et al*., 2013)^. Our laboratory implemented modern approaches to ART treatment and further strengthened to our findings. Finally, and importantly, prior studies utilized older platforms for chromosomal copy number analysis, generally either fluorescence in situ hybridization, SNP array, or aCGH, which are more limited in their ability to analyze the entire complement of chromosomes, to identify smaller deletions and duplications, and to detect mosaicism when compared with NGS.

^Pons *et al*. (2019)^ performed a more recent retrospective study utilizing aCGH to assess fast-cleaving day 3 embryos. Their findings demonstrated that fast day 3 embryos exhibit similar developmental potential to 8-cell day 3 embryos. Our study found similar results and demonstrated improvements in blastulation rate, biopsied blastocyst rate, and euploid rate for every increase in cell count, with a significant difference being our utilization of NGS for PGT-A. Importantly, prior work has demonstrated the improved sensitivity of NGS in detecting lower levels of mosaicism, segmental aneuploidies, and polyploidy when compared to aCGH ^(Friedenthal *et al*., 2018; Werlin *et al*., 2017)^.

Studies utilizing morphokinetics via time-lapse technology have reported similar findings. Campbell *et al.* (2013) found that embryos that reached the compaction and blastulation stages faster were significantly more likely to be euploid than embryos that were delayed at either stage. Similarly, Petersen *et al*. (2016) developed an algorithm to predict blastocyst formation and quality based on morphokinetic data through day 3 of development. In their study, embryos that had ≥ 8 cells on day 3 of development were given a higher score than embryos with < 8 cells. Finally, using time-lapse technology and morphokinetic parameters, Minasi *et al*. (2016) found that the cleavage from three to four cells was significantly faster, the four-cell stage was reached significantly earlier, and blastocysts expanded and hatched significantly faster, in euploid compared with aneuploid blastocysts.

There were several strengths to our study. First, our study was performed at a single academic institution, with a team of uniformly trained embryologists. Second, our study is notable for its large sample size, which allows for greater accuracy and precision and provides more reliable and generalizable results. Last, all PGT-A was performed using NGS, the standard genetic platform for modern ART treatment centers.

This study is not without limitations. The retrospective study design can be subject to selection bias and the potential for confounding variables. Multivariable regression models were utilized to mitigate these effects and accounted for patients who underwent multiple cycles. Additionally, we recognize that the timing of the assessment of day 3 embryos can vary, and this may introduce bias. However, we computed the duration between the time of ICSI and the time of assessment on day 3 and utilized this data to create an adjusted cell count for each embryo, thereby minimizing the effect of this potential variable. Whether through time-lapse technology, non-invasive genomic, transcriptomic, or proteomic correlations, prospective studies should be performed to better delineate the predictive power of day 3 cell count and its relationship with embryonic euploidy.

In conclusion, in the largest study to date evaluating the association between cleavage stage embryo development and embryonic competence, our results demonstrated that cleavage stage embryos with ≥8 cells are more likely to become blastocysts and undergo trophectoderm biopsy than intermediate or slow day 3 embryos. Similar to others, we found that cleavage stage embryos with slow mitotic activity are less likely to develop to the blastocyst stage or undergo trophectoderm biopsy. Our data demonstrated that rapidly dividing cleavage stage embryos have high rates of euploidy and significant developmental potential and perform as well as, if not better than, intermediate or slow growing cleavage stage embryos. We believe that our findings may be applicable in the clinical setting and that patients may be reassured that fast cleaving embryos have a higher likelihood of blastulation and biopsy and equal likelihood of euploidy when compared to slower day 3 embryos. Future studies should aim to identify precise morphokinetic features that, combined with both genomic and non-genomic markers, create multi-dimensional predictive data to power decision support tools that predict implantation, optimize embryo selection, and improve patient outcomes.
